# Supplementary material for: Association of Exercise Intensity with the Prevalence of Glaucoma and Intraocular Pressure in Men: A Study Based on the Korea National Health and Nutrition Examination Survey
Source: J Clin Med. 2022 Aug 12;11(16):4725. doi: 10.3390/jcm11164725 (PMC9409694; doi:10.3390/jcm11164725)
Supplement: Supplementary file 1 [file jcm-11-04725-s001.zip › Table S1.pdf]

Table S1. Baseline characteristics of study subjects.

	Total (n=6,528)	no Exercise (n=651)	Walking only (n=2,337)	Moderate Intensity Exercise (n=1,132)	Vigorous intensity Exercise (n=2,408)	*P*
Age, years	54.16 ± 0.169	55.25 ± 0.568	57.31 ± 0.297	54.38 ± 0.385	51.39 ± 0.200	<0.001
		a	b	a	c	
BMI, kg/m^2	24.08 ± 0.048	23.76 ± 0.155	23.90 ± 0.086	23.88 ± 0.11	24.37 ± 0.075	<0.001
		a	a	a	b	
Waist, cm	85.11 ± 0.146	85.25 ± 0.494	85.34 ± 0.237	84.88 ± 0.326	85.01 ± 0.212	0.587
SBP, mmHg	123.62 ± 0.291	124.49 ± 0.803	124.81 ± 0.455	123.74 ± 0.653	122.45 ± 0.426	<0.001
		ab	a	ab	b	
age adjusted		125.75 ± 0.791	125.33 ± 0.439	125.32 ± 0.632	125.12 ± 0.442	0.917
age and BMI adjusted		126.02 ± 0.771	125.38 ± 0.443	125.53 ± 0.624	124.99 ± 0.43	0.661
DBP, mmHg	80.85 ± 0.201	79.82 ± 0.66	80.14 ± 0.302	80.24 ± 0.422	81.87 ± 0.308	<0.001
		a	a	a	b	
age adjusted		79.11 ± 0.642	79.85 ± 0.291	79.35 ± 0.423	80.37 ± 0.314	0.133
age and BMI adjusted		79.35 ± 0.608	79.9 ± 0.291	79.53 ± 0.413	80.27 ± 0.305	0.358
Glucose, mg/dL	103.01 ± 0.389	101.9 ± 1.134	105.48 ± 0.831	102.65 ± 0.959	101.53 ± 0.525	<0.001
		ab	a	ab	b	
age adjusted		102.53 ± 1.134	105.73 ± 0.814	103.36 ± 0.948	102.74 ± 0.551	0.028
age and BMI adjusted		103 ± 1.125	105.77 ± 0.807	103.69 ± 0.934	102.55 ± 0.534	0.018
HbA1c, %	6.06 ± 0.027	5.92 ± 0.049	6.22 ± 0.052	6.11 ± 0.061	5.94 ± 0.035	<0.001
		a	b	ab	a	
age adjusted		5.97 ± 0.048	6.24 ± 0.05	6.17 ± 0.059	6.04 ± 0.037	<0.001
age and BMI adjusted		5.99 ± 0.049	6.25 ± 0.05	6.19 ± 0.058	6.03 ± 0.036	<0.001
Vitamin D, ng/mL	20.07 ± 0.16	19.61 ± 0.375	19.66 ± 0.225	20.85 ± 0.313	20.17 ± 0.193	0.003
		a	a	b	ab	
		19.86 ± 0.372	19.76 ± 0.222	21.14 ± 0.317	20.66 ± 0.203	<0.001
		19.83 ± 0.372	19.75 ± 0.222	21.13 ± 0.318	20.66 ± 0.203	<0.001
SE Right, D	-0.59 ± 0.033	-0.58 ± 0.109	-0.43 ± 0.06	-0.52 ± 0.072	-0.74 ± 0.048	<0.001
		ab	a	ab	b	
age adjusted		-0.33 ± 0.103	-0.32 ± 0.053	-0.22 ± 0.065	-0.22 ± 0.043	0.464
age and BMI adjusted		-0.33 ± 0.103	-0.32 ± 0.053	-0.22 ± 0.065	-0.22 ± 0.044	0.443
SE left, D	-0.54 ± 0.032	-0.46 ± 0.101	-0.33 ± 0.056	-0.51 ± 0.075	-0.73 ± 0.048	<0.001
		ab	a	ab	b	
age adjusted		-0.18 ± 0.092	-0.23 ± 0.05	-0.2 ± 0.066	-0.19 ± 0.044	0.954
age and BMI adjusted		-0.18 ± 0.092	-0.23 ± 0.05	-0.2 ± 0.067	-0.19 ± 0.044	0.949
IOPmax, mmHg	14.68 ± 0.061	14.38 ± 0.147	14.62 ± 0.090	14.51 ± 0.118	14.86 ± 0.080	0.003
		a	ab	ab	b	
age adjusted		14.34 ± 0.146	14.60 ± 0.089	14.46 ± 0.120	14.76 ± 0.084	0.016
age and BMI adjusted		14.36 ± 0.146	14.60 ± 0.089	14.47 ± 0.119	14.75 ± 0.084	0.031
Glaucoma, %	0.9 (0.12)	2.0 (0.71)	1.1 (0.22)	0.7 (0.27)	0.6 (0.15)	0.012
Alcohol consumption, %						<0.001
None	16.9 (0.53)	19.2 (1.75)	21.5 (1.07)	16.7 (1.35)	12.9 (0.79)	
Moderate	40.1 (0.79)	41.7 (2.52)	36.8 (1.26)	36.4 (1.86)	43.7 (1.22)	
Heavy	42.9 (0.78)	39.0 (2.28)	41.6 (1.34)	46.8 (1.92)	43.3 (1.19)	
Smoking status, %						0.104
Never	15.1 (0.55)	14.1 (1.69)	15.4 (0.95)	15.1 (1.24)	15.2 (0.86)	
Ex-	45.8 (0.81)	41.4 (2.43)	46.6 (1.31)	43.1 (1.88)	47.3 (1.27)	
Current	39.1 (0.80)	44.5 (2.48)	38.0 (1.30)	41.8 (1.86)	37.6 (1.22)	
Monthly household income, %						<0.001
Lowest	15.8 (0.58)	19.9 (1.66)	21.5 (1.05)	15.9 (1.26)	10.3 (0.74)	
Medium-lowest	25.2 (0.79)	30.5 (2.37)	26.9 (1.25)	23.2 (1.59)	23.5 (1.13)	
Medium-highest	28.4 (0.73)	29.7 (2.30)	27.4 (1.24)	26.8 (1.66)	29.5 (1.14)	
Highest	30.6 (0.86)	19.8 (2.01)	24.2 (1.17)	34.0 (1.84)	36.8 (1.34)	
Education level, %						<0.001
≤Elementary school	19.9 (0.66)	31.9 (2.08)	26.0 (1.13)	21.5 (1.49)	11.8 (0.82)	
Middle school	15.8 (0.57)	16.5 (1.80)	18.5 (0.98)	17.0 (1.42)	13.0 (0.83)	
High school	35.5 (0.79)	38.1 (2.54)	31.6 (1.29)	32.3 (1.84)	39.2 (1.25)	
≥College	28.8 (0.91)	13.5 (1.75)	23.9 (1.26)	29.2 (1.92)	35.9 (1.31)	

Intake caffeine, cup/day %						0.351
<1	24.7 (0.70)	24.1 (2.09)	25.5 (1.27)	25.7 (1.70)	24.0 (1.07)	
1	19.7 (0.64)	18.7 (2.03)	20.9 (1.11)	18.6 (1.45)	19.5 (0.99)	
2	23.8 (0.71)	19.9 (2.11)	23.5 (1.24)	23.8 (1.66)	24.8 (1.11)	
≥3	31.8 (0.84)	37.3 (2.54)	30.1 (1.46)	31.9 (1.87)	31.7 (1.24)	
Total energy intake, kcal/d	2326.96±15.316	2250.29±42.606	2196.76±23.698	2372.25±37.957	2427.73±23.133	<0.001
		ab	a	bc	c	
Total protein intake, g/d	82.66 ± 0.696	76.73 ± 1.898	76.13 ± 1.034	83.16 ± 1.668	88.9 ± 1.109	<0.001
		ab	a	b	c	
Total fat intake, g/d	43.09 ± 0.557	40.63 ± 1.611	37.98 ± 0.734	43.07 ± 1.402	47.61 ± 0.936	<0.001
		ab	a	b	c	
Hypertension, %	24.5 (0.65)	22.7 (1.94)	29.2 (1.18)	25.0 (1.57)	21.2 (0.94)	<0.001
Diabetes, %	10.3 (0.43)	9.4 (1.28)	13.4 (0.84)	11.0 (1.18)	7.9 (0.59)	<0.001
Arthritis, %	5.5 (0.31)	7.3 (1.23)	6.2 (0.53)	5.4 (0.72)	4.6 (0.45)	0.038
Cold hands and feet, %	11.8 (0.53)	14.1 (1.71)	12.8 (0.87)	9.6 (1.04)	11.3 (0.79)	0.060
Exercise frequency, times/wk						
Walking activity	3.99 ± 0.041	NA	4.69±0.059	4.09 ± 0.105	4.35 ± 0.058	<0.001
Moderate activity	1.46 ± 0.037	NA	NA	3.53 ± 0.08	2.14 ± 0.052	<0.001
Vigorous activity	1.19 ± 0.030	NA	NA	NA	2.85 ± 0.047	0
Exercise duration, min/wk						
Walking activity	384.51 ± 8.813	NA	450.89 ± 16.187	388.53 ± 25.229	422.1 ± 13.303	0.099
Moderate activity	193.22 ± 8.248	NA	NA	534.19 ± 27.865	255.07 ± 12.681	<0.001
Vigorous activity	144.12 ± 6.27	NA	NA	NA	345.43 ± 13.482	0

Data with the same lowercase letters indicate non-specific differences between groups, while those with different letters are statistically different, based on post hoc test

Data are expressed as the mean ± SE or the percentage (SE)

NA: not applicable
