# Supplementary figures and images for: Association of Exercise Intensity with the Prevalence of Glaucoma and Intraocular Pressure in Men: A Study Based on the Korea National Health and Nutrition Examination Survey
Source: J Clin Med. 2022 Aug 12;11(16):4725. doi: 10.3390/jcm11164725 (PMC9409694; doi:10.3390/jcm11164725)

**Histogram of IOP**

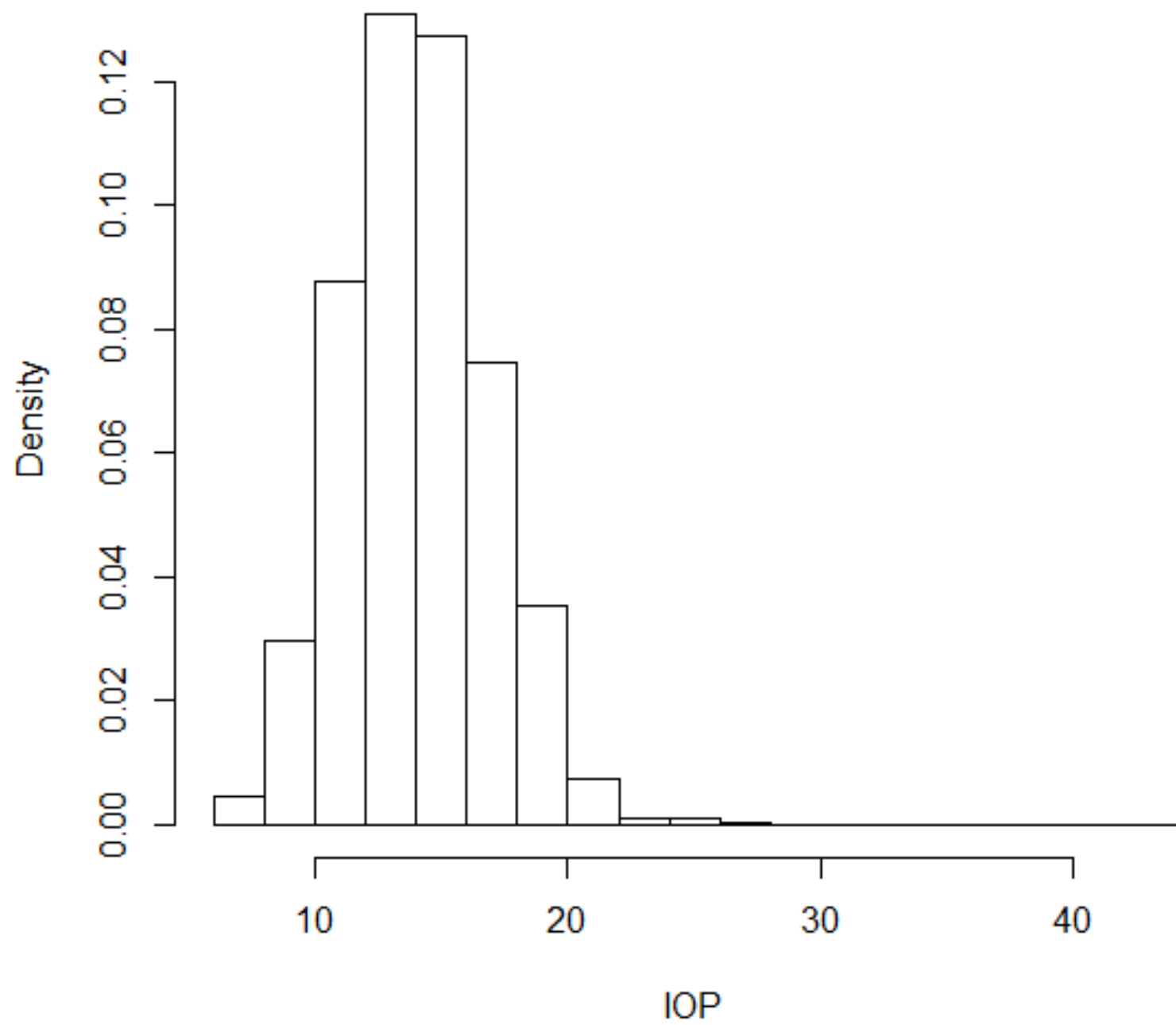

Supplement: Supplementary file 1 [file jcm-11-04725-s001.zip › Figure S1.pdf]
